# The Ecological Significance and Implications of Transovarial Transmission among the Vector-Borne Bunyaviruses: A Review

**DOI:** 10.3390/insects9040173

**Published:** 2018-11-27

**Authors:** Nicholas A. Bergren, Rebekah C. Kading

**Affiliations:** Department of Microbiology, Immunology and Pathology, College of Veterinary Medicine and Biomedical Sciences, Colorado State University, Fort Collins, CO 80523, USA; Rebekah.Kading@colostate.edu

**Keywords:** arbovirus, bunyavirus, *Bunyavirales*, transovarial transmission, vertical transmission

## Abstract

Transovarial transmission (TOT) is a widespread and efficient process through which pathogens can be passed between generations of arthropod vectors. Many species within the order *Bunyavirales* utilize TOT as a means of persisting within the environment when classical horizontal transmission is not possible due to ecological constraints. The purpose of this review is to summarize previous findings regarding the ecological significance of TOT among viruses in the order *Bunyavirales* and identify the gaps in knowledge regarding this important mechanism of arboviral maintenance.

## 1. Introduction

The order *Bunyavirales* comprises the largest RNA virus taxon. This order is subdivided into nine individual families: *Feraviridae*, *Fimoviridae*, *Hantaviridae*, *Jonviridae*, *Nairoviridae*, *Peribunyaviridae*, *Phasmaviridae*, *Phenuiviridae*, and *Tospoviridae*. Viruses in the order Bunyavirales are enveloped virions ranging from 80 to 120 nm in diameter. Their tripartite genome is negative-, or in some cases ambi-sense RNA, and the three genome segments are termed the small (S), medium (M), and large (L).

While the families grouped in the order *Bunyavirales* are based on morphological and biochemical relationships, the specific ecological niche and the transmission strategy can differ dramatically among the various families and their subsequent genera. For example, viruses within the *Orthohantavirus* genus (family: *Hantaviridae*) infect vertebrates and are primarily transmitted through contact with infectious animal excreta, whereas members of the genus *Tospovirus* are plant viruses biologically transmitted by thrips. Indeed, the order Bunyavirales contains viruses that are transmitted between arthropods and vertebrates (arboviruses), arthropods and plants, vertebrates only, and arthropods only. Viruses in the genera *Orthobunyavirus*, *Orthonairovirus*, and *Phlebovirus* are all transmitted to vertebrates by an arthropod vector and negatively impact human health. Orthobunyaviruses are primarily transmitted by mosquitoes and biting midges, orthonairoviruses by ticks, and phleboviruses by phlebotomine sand flies, with the notable exceptions of Rift Valley fever virus which is transmitted by mosquitoes [1], Severe Fever with Thrombocytopenia Syndrome virus (SFTSV) which is transmitted by ticks [2], and Heartland virus (HARV) which is also transmitted by ticks [3].

Transovarial transmission (TOT), the transmission of an infectious agent from parent to offspring via infection of the developing egg which subsequently results in infectious adult arthropods, is an important transmission mechanism among viruses in the order *Bunyavirales*. While several bunyavirus genera use TOT as an important mechanism to maintain themselves in nature, this review will focus on TOT in genera *Orthobunyavirus* and *Phlebovirus* due to the overwhelming evidence of TOT of viruses within these two genera and the impact these viruses have on human health. The purpose of this review is to summarize the key finding of research performed to-date on TOT and identify critical research gaps important for further understanding the ecological significance of this strategy for arbovirus maintenance and control.

Several terms circulate among literature involving vertical transmission and a proper understanding of them is necessary to communicate ideas clearly within the field. Here we provide a list of relevant terms and their definitions. Vertical transmission: transmission of an agent from parent to progeny regardless of mechanism; transovarial transmission: vertical transmission, from female to progeny, whereby the developing ovum is infected and the agent is present in the interior of the egg; transovum transmission: vertical transmission, from parent to progeny, where the agent is transferred to the developed egg and either the egg is infected during insemination or an immature may be exposed while hatching; transovarial transmission rate: the percent of infected females that are transmitting to their progeny; and filial infection rate: the proportion of infected progeny from a single infected female transmitting virus vertically. Throughout this review we will apply these terms as defined above even if the article uses differing terminology.

Due to the nature of this review organizing its structure based on taxonomic relationships of viruses in the order *Bunyavirales*, it is important to note that the International Committee on Taxonomy of Viruses (ICTV) has recently reclassified the family *Bunyaviridae* to the order *Bunyavirales* [4]. Accompanying this change is a restructuring of previously speciated viruses as strains of the same virus species. This review will treat viruses that were classically recognized as species as individual entities due to significant and important differences in their ecologies [5]; however, we will also note where there is harmony among this new taxonomic organization. We will also employ the classical serogroup distinctions in the review’s organization because it provides a good understanding of relatedness that is not captured in the typical taxonomic classifications.

## 2. Genus *Orthobunyavirus*

### 2.1. California Serogroup

The California Serogroup is one of the most widely studied groups of viruses in the context of TOT. Nearly all members of this serogroup undergo TOT, and is a critical mechanism for member viruses to maintain themselves in nature. Interestingly, because of the diverse ecologies of these viruses and, thus, the specific obstacles they must surmount to persist in nature, the uses of TOT are varied and diverse as well.

#### 2.1.1. La Crosse Virus

La Crosse virus (LACV) circulates in North America and can cause severe encephalitis, particularly among individuals younger than sixteen years of age [6]. LACV is maintained primarily between *Aedes* (*Protomacleaya*) *triseriatus* Say, Thomas mosquitoes, and small mammals [7,8,9,10,11]. The mechanisms and efficiency of LACV transmission horizontally, vertically, and venerally by mosquitoes have been exceptionally well characterized. Foundational studies on the transmission mechanism of LACV by *Ae. triseriatus* provide a model system through which to compare TOT by other virus and vector taxa.

The first evidence for TOT as an overwintering mechanism for LACV was the discovery of virus-positive *Ae. triseriatus* larvae collected in the summer of 1972 in Iowa Co., WI, USA [12]. This finding prompted a series of studies that established TOT of LACV as the primary mechanism of overwintering during its transmission cycle. The studies conducted by Watts et al. (1973) were seminal in answering key questions regarding the cycling of LACV between vertebrate and TOT cycles, and the mechanism through which TOT occurs [13,14]. These studies experimentally demonstrated that (1) F_1_ offspring infected with LACV transovarially were capable of infecting a vertebrate host by bite, and (2) that mosquitoes transmitting LACV horizontally were also transmitting LACV vertically through the interior of the egg [13,14]. Additional supporting evidence confirmed that a proportion of female *Ae. triseriatus* mosquitoes reared from field-collected larvae were naturally infected with LACV and capable of transmitting the virus to a susceptible host [15]. This finding was further corroborated by detection of LACV antigen in the salivary glands of day 1 emerged adults via fluorescent antibody [16]. Additionally, ovaries of parental mosquitoes were positive for LACV antigen via IFA, implicating TOT as a primary driver of vertical transmission [16]. Furthermore, LACV transovarially passaged serially in mosquitoes for eight generations remained infective in one- to two-day-old suckling mice, thereby establishing that LACV could persist in nature for approximately four years or longer in the absence of horizontal transmission and remain capable of infecting vertebrates, thereby reestablishing a horizontal transmission cycle [17]. Collectively, these studies demonstrated the importance of TOT as an efficient mechanism for both enzootic maintenance and overwintering for LACV.

Filial infection rates were found to differ dramatically in *Ae. triseriatus* based on ovarian cycle when mosquitoes were infected via bloodmeal. Upon the first ovarian cycle 0% of larvae were positive for LACV; however, upon the second and third ovarian cycles 43% and 58% of larvae, respectively, were found infected with LACV [18]. The finding that filial infection rates increase suggests that the ecology of LACV relies on TOT. Additionally, the observation of increased rates of infected progeny with successive ovarian cycles was corroborated by demonstrating increased infection rates in ovarian and oviduct tissue following second and third noninfectious bloodmeals after an initial infectious bloodmeal [19]. Moreover, infection of LACV did not have any adverse effects on reproductive capacity in *Ae. triseriatus* as indicated by no significant differences in body size, sex-ratio, hatching success, time to ovarian maturation, or fecundity among transovarially infected and uninfected *Ae. triseriatus* [20]. Understanding the intricacies of virus-vector interactions during these sequential blood feeding cycles, and how mosquito infection with LACV carries no fitness cost while TOT efficiency increases remains an important area of research that does not appear to have been pursued recently.

In terms of developmental associations between *Ae. triseriatus* and LACV in developing mosquito larvae and pupae, no definite organ or germ layer is the sole source of infection [16]. Larvae contained the highest viral loads in the alimentary tract (including salivary glands), followed by ganglia, Malpighian tubules, and muscles. Notably, the salivary glands of larvae were found to be infected as early as the second instar. Pupae showed a similar tissue tropism with virus primarily being found in the foregut; notably virus was also found in gonadal and associated tissues and, as with the larvae, in the developing salivary glands [16]. The presence of virus in the salivary glands during larval and pupal development is critically important, as it supports the findings that mosquitoes are, upon eclosion, immediately capable of transmitting LACV by bite [17]. Furthermore, because virus is already established in germline tissue pre-eclosion very little development would have to occur to facilitate TOT. Mechanisms of establishing an infection that proceeds to TOT within the ovaries and other reproductive tissues during the first ovarian cycle of an infected female warrants further investigation.

Another non-classical means of arbovirus transmission via the venereal route, where an infected male passes the virus to a naïve female. LACV does undergo venereal transmission because males can be infected with LACV as a result of TOT [16]. As such, venereal transmission also plays a role in the maintenance of LACV. Interestingly, rates of venereal infection and resulting oral and TOT transmission rates by female mosquitoes mated to an infected male mosquito were found to be significantly greater if females were infected post-bloodmeal [21]. These data support the idea that establishment of LACV infection and transmission efficiency is associated with the physiological changes taking place during a gonotrophic cycle.

Attempts to determine if transovarial transmission of LACV occurs in other species of mosquitoes have generally been unsuccessful with few exceptions. *Aedes* (*Och*) *atropalpus* (Coquillett) is a rockhole-breeding mosquito normally found in eastern Canada and the eastern United States along the Appalachian range with limited populations in Minnesota, Wisconsin, and Michigan [22], which underwent a significant geographic expansion in the late 1970s and early 1980s into Indiana and Ohio [23], Kentucky [24], and New York [25] via the colonization of discarded tires. This geographic expansion into LACV-endemic regions prompted investigations assessing the vector competence of *Ae. atropalpus* for transmission of LACV. These studies showed similar rates of infection and transmission of LACV by *Ae. atropalpus* after oral exposure when compared to *Ae. triseriatus* [26]. Furthermore, similar rates of TOT were shown between *Ae. triseriatus* and *Ae. atropalpus* after infection via the oral and intrathoracic routes [26]. While *Ae. atropalpus* exhibits attributes in the laboratory that are favorable for being an efficient vector for LACV, no detection of LACV has been made from field-collected *Ae. atropalpus* to date, thus leaving its role as a vector for LACV unknown. Recently, *Ae.* (*Stegomyia*) *albopictus* Skuse was found infected with LACV in Dallas County, Texas indicating a potential expansion in geographic range of LACV via *Ae. albopictus* [27]. A Hawaiian strain of *Ae. albopictus* was also shown to be able to transovarially transmit LACV, albeit only 2.7% of the progeny were positive for LACV [28]. Another study showed TOT rates of *Ae. albopictus*, and *Ae.* (*Stegomyia*) *aegypti* (L.) at 52% and 44%, respectively [29]. The differences in rates of TOT among *Ae. albopictus* between these studies [28,29] is likely explained by variation among the strain of *Ae. albopictus* evaluated. Future studies should map quantitative trait loci and followed by sequencing to determine the genetic pattern of TOT in *Ae. triseriatus*.

The geographic range of LACV extends from the mid-west down the eastern seaboard and into the southern states. While LACV is transmitted primarily by *Ae. triseriatus* throughout its geographic range, the specific ecologies and behaviors of *Ae. triseriatus* can differ in important ways. For example, in Florida *Ae. triseriatus* remains active throughout the year and does not enter diapause, thus having an overwintering strategy is not as critical as it may be in the mid-west. From this difference in geographies the question naturally arises: are *Ae. triseriatus* and LACV from the mid-west particularly adapted to facilitate TOT of LACV, and conversely, is TOT of LACV by *Ae. triseriatus* from the south less efficient? A study attempting to determine potential differences in TOT among *Ae. triseriatus* from Wisconsin and Florida found no significant differences in the rate of TOT between the two mosquito strains [30]. However, comparing TOT rates among *Ae. triseriatus* populations may not be the best means to determine the contribution of mosquito genetic background to the efficiency of TOT of LACV. When TOT in *Ae. triseriatus* is selected against, filial infection decreased from 18% to 3% in only three generations; however, when the reciprocal experiments were conducted (selecting for permissive TOT) no significant change in the average filial infection rate occurred [31]. Additionally, in a follow up study, three quantitative trait loci were found to play a role in the permissiveness of TOT in *Ae. triseriatus* [32]. These studies clearly establish a link between genetic factors within the mosquito and its permissiveness to TOT. Due to the predisposition *Ae. triseriatus* has toward the TOT of LACV, it may be a decent model to study TOT in arboviruses where the enzootic vector is difficult to maintain in a laboratory setting. Additional work designed to determine the role of environmental and viral genetic factors that play a role in TOT should be conducted.

#### 2.1.2. California Encephalitis Virus

California encephalitis virus (CEV) was first discovered in Kern County, California in 1943 and has been isolated or detected in several mosquito species in the genera *Aedes*, *Culiseta*, and *Culex* [33]. CEV cycles among a variety of vector species (discussed below) and vertebrate hosts; vertebrate hosts likely include California ground squirrels (*Spermophilus beecheyi* (Richardson)), but may also include various species of lagomorphs (*Sylvilagus* spp. and *Lepus* spp.) [34]. Evidence of vertical transmission was first found during studies in Butte Lake, Utah where virus isolations were made from *Aedes* (*Ochlerotatus*) *dorsalis* (Meigen) larvae [35]. Furthermore, *Ae.* (*Ochlerotatus*) *melanimon* Dyar and *Culiseta inornata* Felt (host-seeking females, males, and adults reared from field-collected immatures during the summer) tested positive for CEV, providing additional evidence for vertical transmission in nature [36]. Finding evidence of vertical transmission and persistence of CEV through the various life-stages (transstadial transmission) of two disparate mosquito genera (*Aedes* and *Culiseta*) indicates that the virus genetic background is likely a critical factor in determining a virus’ ability to transmit vertically. Moreover, because *Cs. inornata* overwinters during immature life-stages, it is possible CEV could use TOT as a potential overwintering mechanism. However, the authors collected immatures during all months except November, December, January, February, and March and only found immatures infected with CEV during the summer months [35].

The first laboratory experiments conducted to confirm TOT as a potential overwintering mechanism for CEV utilized intrathoracic inoculation as a route of infection for *Ae. dorsalis* and *Ae. melanimon* and subsequent testing of progeny for virus; these experiments showed 16.5% and 18.0% of F_1_ female *Ae. doralis* and *Ae. melanimon*, respectively, were positive for virus [37]. Furthermore, additional experiments specifically attributed the mechanism of vertical transmission of CEV to TOT by treating the eggs from infected females with 1% bleach and 70% ethanol, followed by 10 min rinsing under water, rearing the eggs to adults, and testing the adults for virus [37]. Experiments also showed that the proportion of infected progeny was not affected by fluctuations in ambient temperature during egg storage prior to hatching, nor did virus infection acquired through TOT affect survival rates of embryos, larvae or adults; however, transovarially-infected larvae took longer to develop than uninfected larvae [37]. The gonotrophic cycle of parental female mosquitoes is an important factor when considering the efficiency of TOT, as filial infection rates of CEV in *Ae. melanimon* and *Ae. dorsalis* were highest among the progeny of the first gonotrophic cycle (21.2%) with declining infection rates after each additional gonotrophic cycle (14.2% and 13.2% for the second and third gonotrophic cycles, respectively) [38]. However, it is critical to note that IT inoculation bypasses the midgut infection and escape barriers and provides a disseminated infection immediately after injection. Thus, drawing firm conclusions of declining infection rates in relation to gonotrophic cycle is difficult. Additional studies that infect mosquitoes orally and provide additional noninfectious after oral exposure would give researchers a better understanding of the relationship between gonotrophic cycle and infection rates.

In a study that tested Californian mosquitoes from different ecological zones within California, all mosquitoes showed susceptibility to TOT when inoculated with CEV intrathoracically (as conducted in previous studies); oral infection experiments were also performed on *Ae.* (*Och.*) *squamiger* (Coquillett) and *Cs. inornata*. Comparing minimal filial infection rates (MFIR, calculated from pooled progeny adult mosquitoes) between IT inoculated and blood-fed mosquitoes indicated a significant drop in the proportion of progeny infected in group that received the infectious blood meal as compared to those infected by IT inoculation. Specifically, the MFIR was 1:26 in inoculated *Ae. squamiger* mosquitoes and dropped to 1:245 in those which were blood fed, and from 1:144 in IT inoculated *Cs. inornata* to <1:193 in blood fed *Cs. inornata* [39]. Furthermore, the dissemination rate for blood-fed *Ae. squamiger* and *Cs. inornata* were 13% and 18% [39], respectively, showing that while developing a disseminated infection is obviously crucial, it is not the only factor in determining a mosquito’s ability to transovarially transmit the virus. While these studies indicate that IT inoculation artificially increases the rate of TOT via bypass of the midgut infection and escape barriers, the data obtained in mosquitoes exposed to CEV through an IT inoculation remain ecologically significant as infection and escape of the midgut is not the only factor in determining the ability or efficiency of TOT.

To better understand the genetic determinants of TOT in CEV, eleven CEV strains isolated from *Ae. melanimon* in various localities in California showed different efficiencies of TOT [38]. This study also tested one strain of CEV in several different strains of *Ae. melanimon* and showed differing efficiencies of TOT dependent on the mosquito population. These results demonstrated that by changing the genetic composition of the mosquito or virus, the efficiency of TOT was altered, indicating that both the mosquito and virus engage in a critical genetic synergy for TOT to occur.

For field populations of *Ae. dorsalis*, TOT of CEV seems to be dependent on a subset of the population of infected females to develop stabilized infections where filial infection rates reach 100% and infected progeny also have 100% filial infection rates [40]. When females that produce filial infection rates at 100% were mated with males from a “low transmitting colony” the filial infection rate remained greater than 90% [40]. This could be explained by the hypothesis that TOT of CEV is independent of genetic factors variable within the mosquito population, thus, this transmission mechanism is perpetuated by the stochastic development of a stabilized infection in the germline tissue of the mosquito. However, this study was limited in two ways: (1) the genetic factors controlling development of stabilized infections could be sex-linked, researchers would need to backcross the heterozygote F_1_ females to the homozygous recessive males from the “low transmitting colony” and determine infection persistence to address this issue; and (2) the underlying genetic factors controlling possible stochastic mechanisms that lead to stabilized infection were not truly tested as only the progeny of female mosquitoes that were already stably infected were mated with males from the “low transmitting colony” were tested. It could be the case that once the germline tissue is infected persistence of the infection is independent of the factors determining the formation of a stabilized infection in the context of a disseminated virus infection in the mosquito. Importantly, a similar phenomenon was observed with LACV discussed below [41].

These studies on vertical transmission of CEV are valuable in that field evidence for this phenomenon in both *Aedes* and *Culiseta* mosquitoes is supported by laboratory studies that evaluated the mechanisms and efficiency by which TOT occurs in each of these mosquito genera. However, while field evidence supports species in both genera being involved in maintenance of CEV through TOT, laboratory evidence suggests the relative importance of these mosquito species likely varies between species, as well as the specific ecosystem.

#### 2.1.3. Jamestown Canyon Virus

Jamestown Canyon virus (JCV) occurs throughout temperate North America [42]. The first evidence of vertical transmission is attributed to a study on the epidemiology of LACV in Gambier, Ohio, during which JCV was isolated from adult *Ae. triseriatus* which had been raised from field-collected eggs [43]. Furthermore, JCV has been isolated from adult male *Ae.* (*Och.*) *stimulans* Walker in Northern Indiana [44], immature *Ae.* (*Och*) *cataphylla* Dyar in the Sierra Nevada Mountains, and from *Ae.* (*Rusticoidus*) *provocans* (Walker) and *Ae.* (*Och.*) *abserratus* (Felt and Young) [45], all of which provides strong field evidence that vertical transmission plays an important role in the enzootic maintenance of the virus.

Experimental verification of vertical transmission was demonstrated by intrathoracically inoculating mosquitoes that inhabited alpine, costal, and Central Valley localities in California and measuring the proportion of infected F_1_ adults and/or larvae [46]. Of the alpine mosquitoes tested, *Ae.* (*Och*) *tahoensis* Dyar demonstrated the highest filial infection rate with mean filial infection rates (MeFIR) of 1:4, whereas adult *Ae. cataphylla* demonstrated MeFIR of <1:17. For the costal mosquitoes, *Ae. squamiger* adults showed the highest rates MeFIR of 1:4. However, the infection rate was dependent on larval incubation temperature; when *Ae. squamiger* larvae were reared at 10 °C (normal rearing temperature was 20 °C) the MeFIR dropped to 1:11. Lastly, *Cs. inornata* collected from the Central Valley, demonstrated a MeFIR of 1:4 [46]. A similar effect was observed with *Ae. dorsalis* and CEV, when larval rearing temperatures were decreased a reduction in MeFIR was observed [37]. Although, these experiments, nor any others, tested specifically for TOT by treating the eggs with a disinfectant to eliminate the possibility of transovum transmission, it is extremely likely that TOT is the mechanism of vertical transmission given the nature of the viruses within the California encephalitis serogroup.

#### 2.1.4. Trivittatus Virus

Trivittatus virus (TVTV) was first isolated in Bismarck, North Dakota in 1948 and has since shown to be widely distributed throughout North America particularly in the Mid-West via repeated isolations from *Ae. trivittatus* [47]. While humans are exposed to the virus, as evidenced by serological studies, little is known about the consequences of infection [48]. Evidence of TOT of TVTV in *Ae. trivittatus* was first reported when virus was successfully isolated from 1st brood larvae, supporting TOT as an overwintering mechanism for TVTV [49]. In the laboratory, filial infection rates of orally infected *Ae. trivittatus* females were 19%, 23%, and 10% in larvae, female, and male mosquitoes, respectively [50].

While TVTV falls into the California encephalitis serogroup, it serves as an intermediate between the California encephalitis serogroup and the Bwamba/Pongola serogroup. To date, there is no evidence of TOT occurring among viruses recognized as members of the Bwamba/Pongola serogroup; however, this could be due to a lack of collected data. If indeed members of the Bwamba/Pongola serocomplex are not transovarially transmitted, then comparative genetic studies between TVTV and members of the Bwamba/Pongola serogroup could be useful for elucidating the viral genetic determinants of TOT among the Orthobunyaviruses.

#### 2.1.5. Snowshoe Hare Virus

Evidence of TOT in Snowshoe hare virus (SSHV) was first found in the Yukon Territory of Canada in May of 1974, where an isolate of SSHV was made from an *Aedes* spp. larvae [51].

While this evidence suggests TOT may be an available mechanism of overwintering for the virus, overwintering may be accomplished through other mechanisms or a combination thereof.

For example, the same study conducted an experiment showing that adult female *Cs. inornata* were susceptible to virus infection and demonstrated antigen in their salivary glands out to 138 days while held at 0 °C [51], indicating another possible overwintering mechanism. Additional evidence for TOT of SSHV came in 1976 with the isolation of SSHV from *Ae.* (*Och*) *implicatus* Vockeroth larvae in Saskatchewan [52]. Field isolations of SSHV from multiple species of mosquitoes that inhabit northern latitudes, including from larval stages, is highly suggestive that the enzootic maintenance strategies utilized by SSHV also incorporate vertical transmission by mosquito vectors. It is worth noting that neither of these articles pursued studies where eggs from infected females were surface disinfected and hatched to rule out transovum transmission but given the pattern of TOT among the California serogroup, assumption of TOT is not unreasonable.

In order to assess viral genetic determinants of TOT, *Ae. triseriatus* and *Cs. inornata* were compared in their ability to transmit LACV and SSHV vertically [53]. As expected, LACV was efficiently transmitted vertically with 53% of *Ae. triseriatus* females transmitting the virus transovarially. SSHV was also transmitted vertically by adult *Cs. inornata* females (the presumed primary vector for SSHV) at a rate of 89% (though sample sizes were low with only nine mosquitoes tested). After these baselines were established, LACV/SSHV reassortant viruses were used to determine which virus genome segment(s) governed TOT. Significantly, the results from this experiment showed that viruses containing the M segment of LACV were most efficiently transmitted by *Ae. triseriatus*, indicating that viral genetic determinants critical for TOT are most likely encoded on the M segment [53]. The significance of the role of the M segment is consistent among findings that indicate that the NSm gene of Rift Valley fever virus is critical in allowing for virus replication and dissemination from the midgut of *Aedes aegypti* mosquitoes [54]. Notably, the mosquitoes infected with reassortant viruses were inoculated via the intrathoracic route indicating that genetic determinants on the M segments, likely NSm, play a critical role beyond facilitating midgut infection and dissemination, including infection of ovarian tissue and, subsequently, developing eggs. This hypothesis was further supported by a study that reared field collected *Ae. triseriatus* eggs and classified subsets of the collected mosquitoes as super-infected (containing infectious virus and large accumulations of viral antigen and RNA), infected (no detectable infectious virus and lesser amounts of viral antigen and RNA), and non-infected (no detectable infectious virus, antigen, or RNA) [41]. The study found that the NSm gene differed by four amino acid changes from the super-infected subset as compared to the other subsets. The results from these studies show that virus evolution plays a significant role in the development of efficient TOT and is likely specific based upon the enzootic vector(s) the virus co-evolved with.

#### 2.1.6. San Angelo Virus

San Angelo virus (SAV) was originally isolated in Texas from a pool of *Anopheles pseudopunctipennis* Vargas mosquitoes in 1958 [55]. To date, no field evidence of TOT has been presented for SAV; however, experimental TOT of SAV among *Ae. albopictus* has become a crucial model system for elucidating the traits and mechanisms of TOT among bunyaviruses. Initial studies sought to determine which mosquito species were capable of transovarially transmitting SAV. *Aedes* (*Adm*) *vexans* Meigen (Kahua strain), *Culex* (*Cx.*) *quinquefasciatus* Say (Kilihau strain), and *Toxorhynchites amboinensis* Doleschall (Oahu strain) were all refractory to TOT, only *Ae. albopictus* was able to vertically transmit the virus [56]. To characterize intraspecies variation in TOT efficiency, populations of *Ae. albopictus* collected from Taipei, Pontianak, Hong Kong, Oahu, Chon Buri, Korea, and Tokyo were assessed for their capacity to transmit SAV vertically. All populations yielded a percentage of infected progeny between 22.7% and 8.0%, except for the Tokyo population which had a 2.7% progeny infection rate [56]. Overall, all populations of *Ae. albopictus* tested showed a certain degree of permissiveness to TOT. Furthermore, serial passage of SAV via TOT did not significantly affect the rate of TOT, nor did drying the eggs and holding them for three months at 28 °C or altering the larval rearing temperature (held at various gradations between 20 and 32 °C). Additionally, no difference in development rate was found between the progeny of infected and uninfected parents [56].

Further studies were conducted in order to determine the general mechanism of TOT of SAV by *Ae. albopictus.* To determine inheritance patterns of TOT, a colony of *Ae. albopictus* was selected such that the TOT rate and FIR both approached 100% [57]. Maternal inheritance was determined to be the mechanism of TOT, as the infection status of the parent male was of no consequence. One important insight resulting from these studies was that the transmission pattern of SAV in *Ae. albopictus* was similar to that of sigma virus in *Drosophila melanogaster*, and the authors hypothesized that some *Ae. albopictus* females that were selected for their ability to transmit SAV transovarially developed a chronic infection in their germinal cells [57], not unlike the similar observation with CEV [40]. To follow up on this hypothesis, ovaries from chronically-infected *Ae. albopictus* were examined by immunofluorescence assay (IFA) in order to determine the tissue tropism of SAV during various stages of development in the mosquito [58]. Viral antigen was not seen in the germarium at any stage of ovarian development. Though, antigen was found in the follicular epithelium, nurse cells, and oocytes (which all arise from the germarium) during the second and third stages of ovarian development. Antigen was found in the oviduct and ovariole sheath immediately after emergence, indicating that infection does not initiate in the germ cells, although it is possible that SAV is present in the germ cells at undetectable levels. Notably, after female mosquitoes imbibed a blood meal, a rapid accumulation of viral antigen was observed in developing oocytes. The best supported hypothesis for the sequence of infection that allows for TOT of SAV by *Ae. albopictus* is that the virus enters the oocyte through the follicular epithelium from surrounding non-ovarian structures, which is a common mechanism used for other transovarially transmitted endosymbionts of insects [59,60].

#### 2.1.7. Tahyna Virus

Much like the case for other orthobunyaviruses, evidence for TOT of Tahyna virus (TAHV) came from the isolation of virus from field collected *Culiseta annulata* (Schrank) larvae in south Moravia in 1974 [61]. One study that assessed the ability of *Aedes* (*Och*) *caspius* (Pallas) to transovarially-transmit TAHV failed to demonstrate infection of any F_1_ generation mosquitoes [62]. However, laboratory demonstration of TOT was shown with *Aedes vexans* Meigen [63]. Additionally, *Ae. aegypti* was shown to be permissive to TOT of TAHV [64].

#### 2.1.8. Keystone Virus

Keystone virus was shown to transmit vertically in *Ae.* (*Och*) *atlanticus* Dyar and Knab mosquitoes through field collections during 1972 and 1973 on the Delmarva Peninsula, located on the eastern seaboard of the United States [65]. *Ae. atlanticus* females lay their eggs in depressions that are periodically flooded during high rainfall events. Field efforts were organized around rainfall events to maximize collection of larvae post-rainfall. Larvae were either tested or reared to adults and then tested. Results yielded an infection rate of 1:518 in field collected larvae and infection rates ranging from 0:660 to 1:345 in field collected larvae raised to adult females (the disparity in infection rates is due to a difference in sampling sites) [65].

### 2.2. Bunyamwera Serogroup

Only two viruses within the Bunyamwera serogroup have been assessed for their ability to undergo TOT: Cache Valley virus (CVV) and Northway virus (NORV), both in *Cs. inornata* (a laboratory colony for the CVV study and field caught immatures for the NORV study). These studies showed very low rates of TOT, CVV had a TOT rate of 0.15% to 0.40% and NORV showed a FIR of 1:248 [66,67]. Field evidence for TOT among members of the Bunyamwera serogroup is lacking and should be further studied to better understand their ecology.

### 2.3. Simbu Serogroup

Much investigation remains to be completed for viruses in the Simbu serogroup. However, noteworthy field evidence for TOT exists for a couple of viruses within this group.

#### 2.3.1. Akabane Virus

Akabane virus (AKAV) was first isolated from *Ae. vexans* and *Cx.* (*Cux*) *tritaeniorhynchus* Giles mosquitoes in 1959 in the village of Akabane (and other villages) of the Gunma Prefecture, Japan [68]. While adult animals do not usually develop symptoms upon infection, AKAV can cause abortions, stillbirth, and congenital deformities in sheep, cattle, and goats [69]. Although these first isolations were from mosquitoes, the primary vector of AKAV appears to be biting midges in the genus *Culicoides* (consistent with other members of the Simbu serogroup), and experiments that implicate mosquitoes as a vector for AKAV simply have not been conducted. However, several subsequent studies have implicated several *Culicoides* species as vectors for AKAV [70]. While it is possible that mosquitoes act as a biological vector for AKAV, it is also possible that researchers only collected engorged mosquitoes that recently fed on a viremic host. More studies are needed to implicate mosquitoes in the transmission of AKAV. Attempts have been made to investigate TOT as part of the natural transmission cycle of AKAV by collecting and testing immature *C. brevitarsis* in Australia, but no successful isolations have been made to date [71]. Akabane virus was also once considered a select agent with USDA-APHIS, but was removed from the list in 2012 along with several other animal pathogens. Select agent status creates additional biosafety challenges and considerations for working with pathogens under this classification and could be one reason why a significant amount of work has not been conducted with this virus. Future research should revisit the role of mosquitoes as vectors of this virus, particularly those that utilize livestock as blood hosts, and experimentally test the efficiency of TOT by key vector groups.

#### 2.3.2. Schmallenberg Virus

Schmallenberg virus (SBV) was recently discovered in 2011 during outbreak in Europe [72]. As with most members of the Simbu serogroup, the primary vectors are *Culicoides* midges [73]. Evidence of TOT for SBV came from field collections of midges that were separated into pools based on species and parity status [74]. The study used a duplex qRT-PCR for the detection of SBV S segment and 18S gene fragments. Among nulliparous (not having developed eggs yet) midges, the infection rate was 10.8% [74]. While, this relatively high rate of infection among newly-emerged midges is highly suggestive that some vertical transmission mechanism may play a role in the maintenance of SBV, qRT-PCR does not confirm the presence of virus, it only confirms the presence of viral RNA and should be confirmed via plaque assay.

## 3. Genus *Phlebovirus*

### 3.1. Rift Valley Fever Virus

Rift Valley fever virus (RVFV) is a human and veterinary pathogen transmitted by mosquitoes. RVFV is endemic to Africa and has been associated with large outbreaks of severe disease in parts of Africa and the Arabian Peninsula [75]. A main characteristic of RVFV outbreaks is extensive mortality and abortion among infected livestock animals [76]. Human cases arise from either the bite of an infected mosquito or contact with contaminated livestock tissues. While the majority of human cases are self-limited, a small portion can progress to more severe disease manifestations including hepatitis, retinitis, or delayed onset encephalitis. Additionally, severe symptoms can progress to hemorrhagic manifestations which result in a high case fatality [76]. RVFV is a particularly important pathogen due to its proven ability to expand its geographic range and cause epizootic events in geographically naive areas. For this reason, understanding the transmission cycle of RVFV in areas where it is endemic and potential transmission cycles in areas where it hasn’t yet reached is of critical importance for the control and prevention of this arbovirus.

The transmission cycle of RVFV is closely tied to its environment, with TOT supposedly playing a critical role in maintenance of the virus between inter-epizootic periods (IEPs). Periods of high virus activity are closely correlated with periods of heavy rainfall, with El Nino Southern Oscillation being a likely indicator (about every six years), likewise IEPs are correlated with periods of low-to-normal rainfall [77,78]. This correlation of virus outbreaks with rainfall is due to mass emergences of floodwater *Aedes* spp. mosquitoes occurring in low-lying areas, dambos, where eggs had been laid, awaiting a flood event to trigger hatching. Evidence that TOT plays a crucial role in the enzootic maintenance of RVFV was obtained when RVFV was isolated from adult male and female *Ae.* (*Neomelaniconion*) *mcintoshi* Huang (formerly recognized as *Aedes lineatopennis*) mosquitoes that were reared from field-collected larvae and pupae in Kenya during an IEP [79]. During periods of heavy rainfall, the dambos are flooded into their vegetated periphery where the floodwater *Aedes* spp. mosquitoes lay their eggs. The infected eggs hatch and develop resulting in a bloom of infectious floodwater *Aedes* spp. mosquitoes. This bloom of infectious mosquitoes then precipitates an epizootic which involves wild vertebrate hosts, ruminants, humans, and *Culex* spp. and other mosquitoes acting as spillover vectors [80]. The infectious floodwater *Aedes* spp. mosquitoes then oviposit their infected eggs in the same dambos which, when flooded, will result in another epizootic event, thus perpetuating the transmission cycle.

Apart from the evidence provided by the field collections in the 1980s, little more has been done to understand TOT of RVFV. This lack of research is mostly due to the inability to generate a stable colony of floodwater *Aedes* spp. mosquitoes from Africa with which to conduct the research [81]. Many studies have been conducted to assess the vector competence of mosquitoes from non-endemic areas for RVFV, which was recently reviewed [82]. Several North American mosquito species were emphasized as having a high potential for efficient RVFV transmission: *Ae.* (*Och*) *canadensis* Theobald, *Ae.* (*Fin*) *japonicus japonicus* (Theobald) *Ae.* (*Och*) *taeniorhynchus* (Weidemann) *Coquillettidia* (*Cq*) *perturbans* (Walker) *Cx.* (*Cux*) *pipiens* L., *Cx* (*Cux*) *tarsalis* Coquillett, and *Psorophora* (*Jan*) *ferox* (Von Humboldt)*.* However, none of the studies that assessed the vector competence of these mosquito species determined if TOT was a potential aspect of their transmission of RVFV [83,84,85,86]. Turell and colleagues (1990) did assess transstadial transmission of RVFV by exposing *Cx. pipiens*, *Ae. circumluteolus*, and *Ae. mcintoshi* to RVFV as larvae via the oral route, the *Cx. pipiens* were derived from a colony and the *Aedes* spp. mosquitoes were collected as eggs from dambos in a RVFV endemic area [87]. Following exposure to RVFV the larvae yielded infection rates of 9% for *Cx. pipiens* and 8% for the *Aedes* spp. mosquitoes (they were only identified to the subgeneric level). Greater than 1000 adults, reared from the same *Aedes* spp. eggs, were not positive for RVFV indicating the likelihood that the eggs tested were not infected prior to the RVFV treatment as larvae. Larvae were also subjected to differing incubation temperatures and found higher rates to infection in 22 °C water than 30 °C water.

Determining the efficiency by which RVFV can be maintained vertically by African as well as North American mosquito species is critically important to understanding how this virus is maintained in areas of endemicity, as well as how it might establish in more temperate vector populations in the event of an introduction. Furthermore, a similar undertaking should be taken for potential introductions of RVFV in South America with a focus on *Sabethes* in addition to *Aedes* and *Culex* mosquitoes [88].

### 3.2. Sand Fly-Borne Phleboviruses

Compared to other arboviruses transmitted by ticks and mosquitoes, the sand fly-borne viruses in the genus *Phlebovirus* are relatively understudied and much of the specific information regarding TOT come from the same publications. For this reason, we have summarized pertinent research findings under one heading rather than dividing the group into its individual viruses. The majority of phleboviruses are transmitted by phlebotomine sand flies and cause a disease collectively referred to as Phlebotomus fever. Presumptive evidence of vertical transmission of phlebotomus fever by sand flies, or “sandfly fever virus” was first reported by Whittingham and again by two Russian groups in 1924, 1937, and 1938, respectively [89,90,91].

One study fed *Phlebotomus papatasi* Scopoli on patients with sandfly fever, no later than 20 h after onset of disease symptoms, and collected the resulting eggs and reared them to adults [89]. The resulting F_1_ generation was allowed to feed on human volunteers in an attempt to reproduce the disease; as a control, a group of volunteers were inoculated with the blood of the patients the parental sandflies were allowed to feed upon. As would be expected, the control volunteers all produced infection, defined by febrile symptoms, and volunteers receiving bites from the F_1_ sand flies experienced disease symptoms, though development of infection appears to be dependent on the amount of time between egg laying and infective blood feeding of the parental generation [89]. The specific viruses tested in these experiments are unknown because serological techniques to identify the virus were not yet developed.

TOT among sand fly-borne phleboviruses is critical to the maintenance of these viruses in nature. Phlebotomine sand flies ingest a minute quantity of blood when they feed (0.3–0.5 µL), which means that in order to ingest one virus particle, the viremia in the vertebrate host must be at least 4 log_10_ PFU (plaque forming units)/mL [92,93], though one infectious particle doesn’t necessarily correlate to one PFU. These physical constraints present a selective advantage to the virus for an alternative mechanism of transfer between flies. In an experiment where volunteers were infected with a virus isolate of the Sicilian serotype of sandfly fever virus, the viremia lasted less than 48 h and never exceeded 3.4 log_10_ PFU/mL, making the probability of infection by sand flies feeding on a viremic humans extremely low [93,94]. Furthermore, under laboratory conditions, Toscana virus was able to be maintained vertically for 15 generations of *Phlebotomus perniciosus* Newstead without any significant reductions in filial infection rate [95]. Field collections have also indicated TOT to be an important mechanism among these viruses. Isolations from adult male sand flies have been made for Punta Toro, Aguacate, Cacao, Sicilian, Karimabad, Pacui, Ariba, and Toscana viruses [96,97,98,99]. Systematic studies to understand each individual virus’ efficiency at TOT were conducted; the results are summarized in Table 1.

### 3.3. Severe Fever with Thrombocytopenia Syndrome Virus

Severe fever with thrombocytopenia syndrome virus is a newly discovered *Phlebovirus* that causes a febrile illness with thrombocytopenia and leukocytopenia and has a case fatality rate between 10% and 30% [103,104]. The virus was initially found in China [103], but has now been found in South Korea [105] and Japan [106]. Studies characterizing the ecology of SFTSV have detected the virus in *Haemaphysalis longicornis*, Neumann the longhorned tick, thus incriminating it as a potential vector [103,107]. Importantly, *H. longicornis* has been introduced to the US and is now in several states [108,109,110].

A recent report sought to determine if *H. longicornis* ticks were capable of transmitting SFTSV through both vertical and transstadial routes [111]. Female *H. lonicornis* ticks were injected with SFTSV or PBS and, following 12 days post-infection, ticks were allowed to feed on Balb/C mice and maintained for egg laying. Eggs from infected females were separated so that each group of eggs belonged to one infected female; subsequently, a proportion of eggs, larvae, engorged larvae, nymph, engorged nymph, male adult, female adult, female hemolymph, male hemolymph, and male saliva were all tested in pools of varying sizes. All parental ticks were positive for SFTSV indicating successful inoculation and infection. Importantly at least a portion of every egg lay was positive for virus, indicating that the rate of TOT approaches 100%. However, a decrease in infected females and males is observed as the ticks progressed through their various life stages (44.0% and 36.0% of adult female and male pools positive for virus), indicating that transstadial transmission barriers are present. This study assessed infection status of ticks via qRT-PCR. Although the researchers did not test for infectious virus in the ticks, seroconversions of Balb/C mice after being fed upon by the ticks supports that these results represent actual transmission events.

### 3.4. Heartland Virus

Heartland virus (HRTV) was initially isolated from two adult male patients that were hospitalized due to severe febrile illness in northwestern Missouri [112]. Due to the discovery, additional cases have been reported, both fatal and non-fatal [113,114]. The two adult males, from whom the virus was initially isolated, reported being bitten by ticks the week prior to symptom onset, thus prompting field studies to identify potential vector species of the virus. These studies showed that only *Amblyomma americanum* (L.) were positive for HRTV, all other ticks and mosquitoes tested were negative [3].

With *A. americanum* incriminated as a vector for HRTV, a series of laboratory experiments were conducted to assess the virus’ ability to undergo vertical and transstadial transmission [115]. To assess transstadial transmission, *A. americanum* tick larvae were immersed in a HRTV suspension and then fed on a rabbit. Engorged larvae were allowed to molt to nymphs; 39% of nymphs were positive for the virus. Remaining nymphs were fed on a rabbit and progressed to adults, adults were fed on a rabbit again and eggs were collected. Remaining parental adults were tested for virus yielding an infection rate of 54% and 33% in female and male ticks. Larvae from the parental females were then tested for HRTV, of the 11 virus-positive females that laid eggs only five had larvae test positive for the virus, indicating a TOT rate of 45% (5/11). Larvae were tested in pools, which makes FIR difficult to determine; however, a majority of the pools tested from virus-positive females were positive for virus, indicating that FIR may be high.

## 4. Conclusions

Through this literature review we have collectively shown that TOT among viruses in multiple genera of the order *Bunyavirales* is common, and appears to be an extremely important aspect of their maintenance in nature. We have described many representative viruses within the genera *Orthobunyavirus* and *Phlebovirus* which undergo vertical transmission in a variety of arthropod vectors including mosquitoes, sandflies, and ticks. Additional examples of TOT also exist for the nairoviruses and tospoviruses which are not covered in this review. However, despite the ecological significance of TOT, research into this mechanism has declined after the phenomenon was established in the 1970s and 1980s. Few studies have sought to understand the underlying genetic determinants (on the part of the vector and the virus), biological mechanisms, and evolutionary implications of TOT, apart from some excellent work with SAV and LACV [31,32,56,57,58]. Important questions remain regarding TOT among bunyaviruses as a group and for specific viruses within the order. With the ability to conduct experiments utilizing modern molecular and computational techniques it is now possible to have a much more detailed and mechanistic understanding of this important transmission mechanism. A useful means of synthesizing this data is presented in Table 2, where viruses are categorized against experimentally elucidated determinants of TOT.

**Figure 1:**
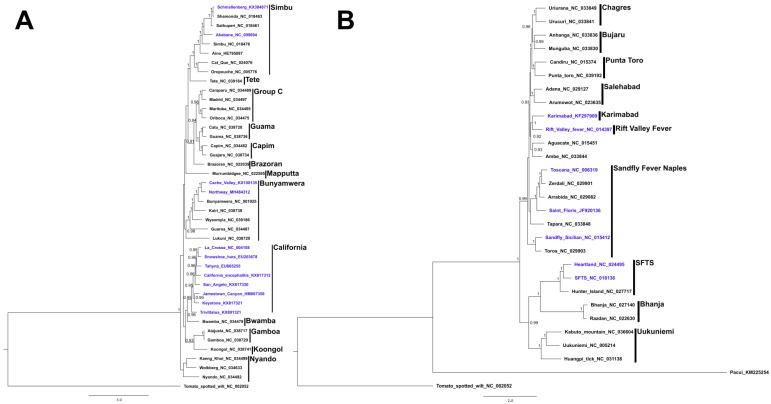
Bayesian phylogenetic trees based on nucleotide sequence of the RDRP for (**A**) orthobunyaviruses and (**B**) phleboviruses. In addition to viruses discussed in the paper available on GenBank, Orthobunyavirus, and *Phlebovirus* sequences available via RefSeq were included in the alignment. Nucleotide sequences were aligned by translating to amino acid, aligning using MUSCLE [116], and back translating to nucleotide. Columns in the alignment were removed where gaps contributed to 80% of the column composition using Trimal [117]. Substitution models were chosen with jModelTest2 (GTR Γ + I for both phylogenies) [118]. Trees were generated in Mr. Bayes [119] with 5,000,000 steps, sampling every 1000 and discarding the first 10% as burn-in. Convergence was assessed by examining the stationary ln-likelihood and effective sample size (ESS, >200) parameters in Tracer v1.7.1 (BEAST, Auckland, New Zeland). Posterior probabilities reported on the trees are >0.9.

One important aspect toward a more comprehensive knowledge of TOT is understanding its evolutionary origins. TOT appears to be quite ubiquitous among the bunyaviruses and understanding whether TOT arose once or convergently during its evolutionary history would be informative. This information would be useful in understanding the specific ecological constraints that must be in place to allow for TOT to be selected. Many studies have shown that the relationship between virus and vector is critical for efficient TOT, this seems to be suggestive that TOT is a result of convergent evolution and the surrounding ecology puts significant pressure on the virus to develop a means of TOT. Figure 1 provides maximum likelihood phylogenies based on the amino acid sequences of the RNA-dependent RNA polymerase (RDRP) for orthobunyaviruses and phleboviruses. These phylogenies seem to provide further support to the convergent evolution hypothesis due to TOT appearing to arise from disparate; however, more robust analyses need to be conducted. Many examples of TOT involve viruses operating in temperate geographic areas where continual horizontal maintenance is not possible due to winter months, thus the virus must develop a means of overwintering. While TOT is not the only overwintering mechanism available, it seems to be one that is readily used among the bunyaviruses. Furthermore, there may be some useful insights gained from studying ancestral reconstructions from groups of viruses that have evidence of TOT, such as the California serogroup.

An important gap that exists in our understanding of TOT is understanding the viral genetics that underlie the process. Kading et al. (2014) found that when the NSm gene was deleted from the M segment of RVFV vector competence was significantly reduced. Virus was prevented from entering, replicating in, and escaping from midgut epithelial cells [54]. While wholesale deletion of the NSm gene obviously cripples the virus’ ability to infect and disseminate within the mosquito, there may be genetic determinants within the NSm gene that are specific to infection of the ovaries. Furthermore, this correlates nicely with an earlier study observing recombinant LACV/SSHV and their ability to undergo TOT, this study showed that the M segment was a critical determinant in the virus’ ability to undergo TOT [53]. These two studies clearly show that experiments assessing genetic determinants of TOT should be focused, at least in part, on the M segment. Additionally, having a comprehensive understanding of the evolutionary origins and genetic determinants of TOT would provide important insights regarding other groups of arboviruses that do not utilize TOT as a transmission mechanism.

Another critical gap involves elucidating the mechanism of TOT. Understanding the means by which virus enters the ovaries may provide useful avenues of developing either transgenic mosquitoes refractory to TOT or other countermeasures. Tesh et al. (1981) observed some key features of ovarian infection in bunyaviruses. It appears that virus enters the ovaries via the oviduct and ovariole sheath and subsequently tissues derived from the germarium become infected, including the follicular epithelium, oocytes, and nurse cells of primary follicles. Importantly a drastic increase in virus antigen and progression from the ovariole sheath and oviduct to the germarium-derived tissues of primary follicles was observed upon ingestion of a bloodmeal [58]. Bloodmeals precipitate several metabolic processes that culminates in oviposition, one of which is vitellogenesis. Vitellogenesis is a cornerstone of the reproductive cycle and is the process that generates and deposits massive quantities of yolk protein precursors in developing oocytes [120]. Tesh et al. (1980) observed a significant amount of viral antigen in the fat body, where yolk protein precursors are synthesized, and deposited throughout the yolk of infected oocytes. This correlation suggests that vitellogenesis may play an important role in facilitating the infection of germarium derived tissues, possibly facilitating entry of viral antigen.

It should be noted TOT rates and FIR can drastically differ from one virus to another throughout the bunyaviruses. Additionally, the pairing between virus and mosquito species is critical for efficient TOT. These trends support the idea that while mechanisms may generally be consistent throughout the bunyaviruses, there may be important differences for specific virus-vector interactions that exist because of the ecology of the virus and vector and their evolutionary relationships. However, this is not to dismiss the use of model systems. Important baseline information can be collected from model systems, such as the well-established SAV and *Ae. albopictus* or LACV and *Ae. triseriatus* models. Further studies looking at the mechanism of TOT in the context of a specific virus-vector interaction can utilize these baseline model systems as a starting place in terms of generating a variety of hypotheses geared at determining the mechanism of TOT for the virus and vector being tested and the associated costs of TOT for said virus and vector.

Research on the TOT of RVFV is one area that is lacking significantly when compared to the relative importance of the pathogen. Apart from linking the periodicity of RVFV outbreaks to the flooding of dambos and the subsequent bloom of transovarially-infected floodwater *Aedes* spp. no experiments have been conducted with either African or local vector mosquitoes to better understand the efficiency or mechanisms for the potential vertical transmission of this important emerging arbovirus. Granted, a large contributing factor to this dearth of understanding lies in the difficulty of starting and maintaining a colony of ecologically-relevant floodwater *Aedes* spp. mosquitoes, and available facilities in which to conduct these experiments with a select agent virus. However, more research focus on potential maintenance mechanisms of RVFV and other emerging viruses is needed to pave the way for pursuing molecular and ecological strategies that target mosquito-virus and mosquito-host interactions and prevent transmission to humans.

TOT remains a critical component of many bunyavirus natural transmission cycles. Having a comprehensive understanding of TOT could lead towards more effective control strategies, a better understanding of the evolutionary pressures that determine an arbovirus’ transmission cycle, and a better understanding of the risk of potential introduction events to naive geographic areas.

## Figures and Tables

**Table 1:** Filial infection rates of sand fly-borne Phleboviruses.

Virus	Vector Species	Percent of F_1_ Progeny Infected	Reference
Arbia virus	*Phlebotomus perniciosus*	20.7	[100]
Karimabad virus	*Phlebotomus papatasi*	60.0	[100]
Pacui virus	*Lutzomyia longipalpis*	32.9	[100]
Saint Floris virus	*Phlebotomus papatasi*	6.3	[100]
Sicilian	*Phlebotomus papatasi*	1.5	[100]
Toscana	*Phlebotomus perniciosus*	30.1	[100]
Arboledas virus	*Lutzomyia gomezi*	80.0	[101]
Rio Grande virus	*Lutzomyia anthophora*	54.8	[102]

**Table 2:** Summary of known drivers of TOT efficiency with exemplary virus/vector pairs.

Driver	Virus	Vector	Reference
**Vector influences on TOT**			
Gonotrophic cycle	SAV	*Ae. albopictus*	[56]
	CEV	*Ae. melanimon* *Ae. dorsalis*	[38]
Venereal transmission	LACV	*Ae. triseriatus*	[16,21]
Survival and development time	CEV	*Ae. melanimon* *Ae. dorsalis*	[37]
	SAV	*Ae. albopictus*	[56]
Transmission barriers; vector competence	CEV	*Ae. dorsalis*	[40]
	LACV	*Ae. triseriatus*	[41]
	RVFV	*Cx. pipiens* *Ae. circumluteolus* *Ae. mcintoshi*	[87]
	SFTSV	*H. longicornis* (tick)	[111]
	HRTV	*A. americanum* (tick)	[115]
	SAV	*Ae. albopictus*	[58]
	LACV	*Ae triseriatus*	[16]
	CEV	*Ae. melanimon*	[38]
Quantitative trait loci	LACV	*Ae. triseriatus*	[31,32]
Maternal inheritance	SAV	*Ae. albopictus*	[57]
**Viral influences on TOT**			
M segment critical for TOT	LACV	*Ae. triseriatus*	[53]
NSm deletion	RVFV	*Ae. aegypti*	[54]
Amino acid residues in NSm	LACV	*Ae. triseriatus*	[41]
**Environmental influences on TOT**			
Persistence through interepidemic periods	RVFV	*Ae. mcintoshi*	[79]
Water temperature	JCV	*Ae. squamiger*	[46]
	SAV	*Ae. albopictus*	[56]
Climate patterns/El Nino	RVFV	*Ae. mcintoshi*	[78]

## References

[1] Smithburn K.C., Haddow A.J., Gillett J.D. (1948). Rift Valley fever. Isolation of the virus from wild mosquitoes. Br. J. Exp. Pathol..

[2] Yun S.-M., Lee W.-G., Ryou J., Yang S.-C., Park S.-W., Roh J.Y., Lee Y.-J., Park C., Han M.G. (2014). Severe fever with thrombocytopenia syndrome virus in ticks collected from humans, South Korea, 2013. Emerg. Infect. Dis..

[3] Savage H.M., Godsey M.S., Lambert A., Panella N.A., Burkhalter K.L., Harmon J.R., Lash R.R., Ashley D.C., Nicholson W.L. (2013). First detection of heartland virus (Bunyaviridae: *Phlebovirus*) from field collected arthropods. Am. J. Trop. Med. Hyg..

[4] Adams M.J., Lefkowitz E.J., King A.M.Q., Harrach B., Harrison R.L., Knowles N.J., Kropinski A.M., Krupovic M., Kuhn J.H., Mushegian A.R. (2017). Changes to taxonomy and the International Code of Virus Classification and Nomenclature ratified by the International Committee on Taxonomy of Viruses (2017). Arch. Virol..

[5] Blitvich B.J., Beaty B.J., Blair C.D., Brault A.C., Dobler G., Drebot M.A., Haddow A.D., Kramer L.D., LaBeaud A.D., Monath T.P. (2018). Bunyavirus taxonomy: Limitations and misconceptions associated with the current ICTV criteria used for species demarcation. Am. J. Trop. Med. Hyg..

[6] Saeed O., Afzal M.R., Ahrar A., Chughtai M., Hassan A., Ishfaq M.F., Lobanova I., Malik M.I., Malik A.A., Qureshi M.A., Qureshi A.I. (2018). Chapter 2—Mosquito-Borne Diseases. Zika Virus Disease.

[7] Moulton D.W., Thompson W.H. (1971). California Group virus infections in small, forest-dwelling mammals of Wisconsin. Am. J. Trop. Med. Hyg..

[8] Pantuwatana S., Thompson W.H., Watts D.M., Hanson R.P. (1972). Experimental infection of chipmunks and squirrels with La Crosse and Trivittatus viruses and biological transmission of La Crosse virus by *Aedes triseriatus*. Am. J. Trop. Med. Hyg..

[9] Thompson W.H., Anslow R.O., Hanson R.P., DeFoliart G.R. (1972). La Crosse virus isolations from mosquitoes in Wisconsin, 1964–1968. Am. J. Trop. Med. Hyg..

[10] Sudia W.D., Newhouse V.F., Calisher C.H., Chamberlain R.W. (1971). California Group arboviruses: Isolations from mosquitoes in North America. Mosq. News.

[11] Wright R.E., DeFoliart G.R. (1970). Associations of Wisconsin mosquitoes and woodland vertebrate hosts. Ann. Entomol. Soc. Am..

[12] Pantuwatana S., Thompson W.H., Watts D.M., Yuill T.M., Hanson R.P. (1974). Isolation of La Crosse virus from field collected *Aedes triseriatus* larvae. Am. J. Trop. Med. Hyg..

[13] Watts D.M., Pantuwatana S., DeFoliart G.R., Yuill T.M., Thompson W.H. (1973). Transovarial transmission of La Crosse virus (California Encephalitis Group) in the mosquito, *Aedes triseriatus*. Science.

[14] Patrican L.A., DeFoliart G.R., Yuill T.M. (1985). La Crosse viremias in juvenile subadult and adult chipmunks following feeding by transovarially-infected *Aedes triseriatus*. Am. J. Trop. Med. Hyg..

[15] Watts D.M., Thompson W.H., Yuill T.M., DeFoliart G.R., Hanson R.P. (1974). Overwintering of La Crosse virus in *Aedes triseriatus*. Am. J. Trop. Med. Hyg..

[16] Beaty B.J., Thompson W.H. (1976). Delineation of La Crosse virus in developmental stages of transovarially infected *Aedes triseriatus*. Am. J. Trop. Med. Hyg..

[17] Miller B.R., DeFoliart G.R., Yuill T.M. (1977). Vertical transmission of La Crosse virus (California encephalitis group): Transovarial and filial infection rates in *Aedes triseriatus* (Diptera: Culicidae). J. Med. Entomol..

[18] Miller B.R., DeFoliart G.R., Yuill T.M. (1979). *Aedes triseriatus* and La Crosse virus lack of infection in eggs of the first ovarian cycle following oral infection of females. Am. J. Trop. Med. Hyg..

[19] Beaty B.J., Thompson W.H. (1978). Tropisms of La Crosse virus in *Aedes triseriatus* following infective blood meals. J. Med. Entomol..

[20] Patrican L.A., DeFoliart G.R. (1985). Lack of adverse effect of transovarially acquired La Crosse virus infection on the reproductive capacity of *Aedes triseriatatus*. J. Med. Entomol..

[21] Thompson W.H. (1979). Higher venereal infection and transmission rates with La Crosse virus in *Aedes triseriatus* engorged before mating. Am. J. Trop. Med. Hyg..

[22] Zavortink T.J. (1972). Mosquito Studies (Diptera, Culicidae) XXVIII: The New World Species Formerly Placed in Aedes (Finlaya).

[23] Restifo R., Lanzaro G. (1980). The occurrence of *Aedes atropalpus* (Coquillett) breeding in tires in Ohio and Indiana. Mosq. News.

[24] Covell C.V., Bnowunu A. (1979). *Aedes atropalpus* in abandoned tires in Jefferson County, Kentuky. Mosq. News.

[25] White D.J., White C. (1980). *Aedes atropalpus* breeding in artificial containers in Suffolk County, New York. Mosq. News.

[26] Freier J.E., Beier J.C. (1984). Oral and transovarial transmission of La Crosse virus by *Aedes atropalpus*. Am. J. Trop. Med. Hyg..

[27] Lambert A.J., Blair C.D., D’Anton M., Ewing W., Harborth M., Seiferth R., Xiang J., Lanciotti R.S. (2010). La Crosse virus in *Aedes albopictus* mosquitoes, Texas, USA, 2009. Emerg. Infect. Dis..

[28] Tesh R.B., Gubler D.J. (1975). Laboratory studies of transovarial transmission of La Crosse and other arboviruses by *Aedes albopictus* and *Culex fatigans*. Am. J. Trop. Med. Hyg..

[29] Hughes M.T., Gonzalez J.A., Reagan K.L., Blair C.D., Beaty B.J. (2006). Comparative potential of *Aedes triseriatus*, *Aedes albopictus*, and *Aedes aegypti* to transovarially transmit La Crosse virus. J. Med. Entomol..

[30] Woodring J., Chandler L.J., Oray C.T., McGaw M.M., Blair C.D., Beaty B.J. (1998). Short Report: Diapause, transovarial transmission, and filial infection rates in geographic strains of La Crosse virus infected *Aedes triseriatus*. Am. J. Trop. Med. Hyg..

[31] Graham D., Holmes J., Higgs S., Beaty B., Black W. (1999). Selection of refractory and permissive strains of *Aedes triseriatus* (Diptera: Culicidae) for transovarial transmission of La Crosse virus. J. Med. Entomol..

[32] Graham D.H., Holmes J.L., Beaty B.J., Black IV W.C. (2003). Quantitative trait loci conditioning transovarial transmission of La Crosse virus in the eastern treehold mosquito, *Ochlerotatus triseriatus*. Insect Mol. Boil..

[33] Hammon W.M., Reeves W.C. (1952). California Encephalitis virus—A newly described agent. Calif. Med..

[34] LeDuc J.W. (1979). Review Article 1: The ecology of California group viruses. J. Med. Entomol..

[35] Crane G.T., Elbel R.E., Calisher C.H. (1977). Transovarial transmission of California encephalitis virus in the mosquito *Aedes dorsalis* at Blue Lake, Utah. Mosq. News.

[36] Reisen W., Hardy J., Reeves W., Presser S., Milby M., Meyer R. (1990). Persistence of mosquito-borne viruses in Kern County, California, 1983–1988. Am. J. Trop. Med. Hyg..

[37] Turell M.J., Reeves W.C., Hardy J.L. (1982). Transovarial and Transstadial Transmission of California Encephalitis Virus in *Aedes dorsalis* and *Aedes melanimon*. Am. J. Trop. Med. Hyg..

[38] Turell M.J., Reeves W.C., Hardy J.L. (1982). Evaluation of the efficiency of transovarial transmission of California encephalitis strains in *Aedes dorsalis* and *Aedes melanimon*. Am. J. Trop. Med. Hyg..

[39] Kramer L.D., Reeves W.C., Hardy J.L., Presser S.B., Eldridge B.F., Bowen M.D. (1992). Vector competence of California mosquitoes for California encephalitis and California encephalitis-like viruses. Am. J. Trop. Med. Hyg..

[40] Turell M.J., Hardy J.L., Reeves W.C. (1982). Stabilized infection of Caliornia encephalitis virus in *Aedes dorsalis* and its implications for viral maintenance in nature. Am. J. Trop. Med. Hyg..

[41] Reese S.M., Mossel E.C., Beaty M.K., Beck E.T., Geske D., Blair C.D., Beaty B.J., Black W.C. (2010). Identification of super-infected *Aedes triseriatus* mosquitoes collected as eggs from the field and partial characterization of the infecting La Crosse viruses. Virol. J..

[42] Grimstad P., Monath T. (1988). California group virus disease. The Arboviruses: Epidemiology and Ecology.

[43] Berry R., Weigert B.L., Calisher C., Parsons M., Bear G. (1977). Evidence for transovarial transmission of Jamestown Canyon virus in Ohio. Mosq. News.

[44] Boromisa R.D., Grimstad P.R. (1986). Virus-vector-host relationships of *Aedes stimulans* and Jamestown Canyon virus in a northern Indiana enzootic focus. Am. J. Trop. Med. Hyg..

[45] Heard P.B., Zhang M., Grimstad P. (1990). Isolation of Jamestown Canyon virus (California serogroup) from Aedes mosquitoes in an enzootic focus in Michigan. J. Am. Mosq. Control Assoc..

[46] Kramer L.D., Bowen M.D., Hardy J.L., Reeves W.C., Presser S.B., Eldridge B.F. (1993). Vector competence of alpine, Central Valley, and costal mosquitoes from California for Jamestown Canyon virus. J. Med. Entomol..

[47] Eklund C., Karabatsos N. (1985). Trivittatus virus. International Catalogue of Arboviruses Including Certain Other Virus of Vertebrates.

[48] Monath T.P.C., Nuckolls J.G., Berall J., Bauer H., Chappell W.A., Coleman P.H. (1970). Studies on Caliornia encephalitis in Minnesota. Am. J. Epidemiol..

[49] Andrews W.N., Rowley W.A., Wong Y.W., Dorsey D.C., Hausler J.W.J. (1977). Isolation of Trivittatus Virus from Larvae and Adults Reared from Field-Collected Larvae of *Aedes Trivittatus* (Diptera: Gulicidae). J. Med. Entomol..

[50] Christensen B.M., Rowley W.A., Wong Y.W., Dorsey D.C., Hausler W.J. (1978). Laboratory studies of transovarial transmission of trivittatus virus by *Aedes trivittatus*. Am. J. Trop. Med. Hyg..

[51] McLean D.M., Bergman S.K.A., Gould A.P., Grass P.N., Miller M.A., Spratt E.E. (1975). California encephalitis virus prevalence throughout the Yukon Territory, 1971–1974. Am. J. Trop. Med. Hyg..

[52] McLintock J., Curry P., Wagner R., Leung M., Iversen J. (1976). Isolation of snowshoe hare virus from *Aedes implicatus* larvae in Saskatchewan. Mosq. News.

[53] Schopen S., Labuda M., Beaty B. (1991). Vertical and venereal transmission of California group viruses by *Aedes triseriatus* and *Culiseta inornata* mosquitoes. Acta Virol..

[54] Kading R.C., Crabtree M.B., Bird B.H., Nichol S.T., Erickson B.R., Horiuchi K., Biggerstaff B.J., Miller B.R. (2014). Deletion of the NSm virulence gene of Rift Valley fever virus inhibits virus replication in and dissemination from the midgut of *Aedes aegypti* mosquitoes. PLoS Negl. Trop. Dis..

[55] Grimes J.E., Garza E.H., Irons J.V. San Angelo virus. Proceedings of the 11th Annual Meeting of the American Society of Tropical Medicine and Hygiene.

[56] Tesh R.B. (1980). Experimental studies on the transovarial transmission of Kunjin and San Angelo viruses in mosquitoes. Am. J. Trop. Med. Hyg..

[57] Tesh R.B., Shroyer D.A. (1980). The mechanism of arbovirus transovarial transmission in mosquitoes San Angelo virus in *Aedes albopictus*. Am. J. Trop. Med. Hyg..

[58] Tesh R.B., Cornet M. (1981). The location of San Angelo virus in developing ovaries of transovarially infected *Aedes albopictus* mosquitoes as revealed by fluorescent antibody technique. Am. J. Trop. Med. Hyg..

[59] Koch A. (1967). Chapter 1—Insects and Their Endosymbionts. Symbiosis.

[60] Lanham U.N. (1968). The Blochmann Bodies: Hereditary Intracellular Symbionts of Insects. Boil. Rev..

[61] Bardos V., Ryba J., Hubalek Z. Isolation of Tahyna virus from *Culiseta annulata (Schrk.)* larvae collected in natural surroundings. Proceedings of the 12th Annual Meeting of the Czechoslovak Society for Microbiology.

[62] Moreau J.P., Bihan-Faou P., Sinegre G. (1976). Tahyna virus transovarial transmission, trials in *Aedes caspius*. Med. Trop..

[63] Danielova V., Ryba J. (1979). Laboratory demonstration of transovarial transmission of Tahyna virus in Aedes vexans and the role of this mechanism in overwintering of this arbovirus [laboratory animals]. Folia Parasitol. (Czechoslov.).

[64] Labuda M., Ciampor F., Kozuch O. (1983). Experimental model of transovarial transmission of Tahyna virus in *Aedes aegypti* mosquitoes. Acta Virol..

[65] Le Duc J.W., Suyemoto W., Eldridge B.F., Russell P.K., Barr A.R. (1975). Ecology of California encephalitis viruses on the Del Mar Va Peninsula II. Demonstration of transovarial transmission. Am. J. Trop. Med. Hyg..

[66] Corner L.C., Robertson A.K., Hayles L.B., Iversen J.O. (1980). Cache Valley virus: Experimental infection in *Culiseta inornata*. Can. J. Microbiol..

[67] Kramer L.D., Hardy J.L., Reeves W.C., Presser S.B., Bowen M.D., Eldridge B.F. (1993). Vector competence of selected mosquito species (Diptera: Culicidae) for california strains of Northway virus (Bunyaviridae: *Bunyavirus*). J. Med. Entomol..

[68] Oya A., Okuno T., Ogata T., Kobayashi I., Matsuyama T. (1961). Akabane, a new arbor virus isolated in Japan. Jpn. J. Med Sci. Boil..

[69] Kurogi H., Inaba Y., Takahashi E., Sato K., Omori T., Miura Y., Goto Y., Fujiwara Y., Hatano Y., Kodama K. (1976). Epizootic congenital arthrogryposis-hydranencephaly syndrome in cattle: Isolation of Akabane virus from affected fetuses. Arch. Virol..

[70] Jennings M., Mellor P.S. (1989). Culicoides: Biological vectors of akabane virus. Vet. Microbiol..

[71] Allingham P.G., Standfast H.A. (1990). An investigation of transovarial transmission of Akabane virus in *Culicoides brevitarsis*. Aust. Vet. J..

[72] Hoffmann B., Scheuch M., Hoper D., Jungblut R., Holsteg M., Schirrmeier H., Eschbaumer M., Goller K.V., Wernike K., Fischer M. (2012). Novel orthobunyavirus in Cattle, Europe, 2011. Emerg. Infect. Dis..

[73] Rasmussen L.D., Kristensen B., Kirkeby C., Rasmussen T.B., Belsham G.J., Bodker R., Botner A. (2012). Culicoids as vectors of Schmallenberg virus. Emerg. Infect. Dis..

[74] Larska M., Lechowski L., Grochowska M., Zmudzinski J.F. (2013). Detection of the Schmallenberg virus in nulliparous *Culicoides obsoletus/scoticus* complex and *C. punctatus*-The possibility of transovarial virus transmission in the midge population and of a new vector. Vet. Microbiol..

[75] Nanyingi M.O., Munyua P., Kiama S.G., Muchemi G.M., Thumbi S.M., Bitek A.O., Bett B., Muriithi R.M., Njenga M.K. (2015). A systematic review of Rift Valley Fever epidemiology 1931–2014. Infect. Ecol. Epidemiol..

[76] Meegan J.M., Bailey C.L., Monath T.P.C. (1988). Rift Valley Fever. The Arboviruses: Epidemiology and Ecology.

[77] Davies F.G., Linthicum K.J., James A.D. (1985). Rainfall and epizootic Rift Valley fever. Bull. World Health Organ..

[78] Linthicum K.J., Anyamba A., Tucker C.J., Kelley P.W., Myers M.F., Peters C.J. (1999). Climate and Satellite Indicators to Forecast Rift Valley Fever Epidemics in Kenya. Science.

[79] Linthicum K.J., Davies F.G., Kairo A. (1985). Rift Valley fever virus (family Bunyaviridae, genus *Phlebovirus*). Isolations from Diptera collected during an inter-epizootic peiod in Kenya. J. Hyg..

[80] Bird B.H., McElroy A.K. (2016). Rift Valley fever virus: Unanswered questions. Antivir. Res..

[81] Pepin M., Bouloy M., Bird B.H., Kemp A., Paweska J. (2010). Rift Valley fever virus (*Bunyaviridae*: *Phlebovirus*): An update on pathogenesis, molecular epidemiology, vectors, diagnostics and prevention. Vet. Res..

[82] Linthicum K.J., Britch S.C., Anyamba A. (2016). Rift Valley fever: An emerging mosquito-borne disease. Annu. Rev. Entomol..

[83] Gargan T.P., Clark G.G., Dohm D.J., Turell M.J., Bailey C.L. (1988). Vector potential of selected North American mosquito species for Rift Valley fever virus. Am. J. Trop. Med. Hyg..

[84] Turell M.J., Britch S.C., Aldridge R.L., Kline D.L., Boohene C., Linthicum K.J. (2013). Potential for mosquitoes (Diptera: Culicidae) from Florida to transmit Rift Valley fever virus. J. Med. Entomol..

[85] Turell M.J., Wilson W.C., Bennett K.E. (2010). Potential for North American mosquitoes (Diptera: Culicidae) to transmit Rift Valley fever virus. J. Med. Entomol..

[86] Turell M.J., Byrd B.D., Harrison B.A. (2013). Potential for populations of *Aedes j. japonicus* to transmit Rift Valley fever virus in the USA. J. Am. Mosq. Control Assoc..

[87] Turell M.J., Linthicum K.J., Beaman J.R. (1990). Transmission of Rift Valley fever virus by adult mosquitoes after ingestion of virus as larvae. Am. J. Trop. Med. Hyg..

[88] Mores C.N., Turell M.J., Dyer J., Rossi C.A. (2008). Phylogenetic relationships among Orthobunyaviruses isolated from mosquitoes captured in Peru. Vector-Borne Zoonotic Dis..

[89] Mochkovski S.D., Diomina N.A., Nossina V.D., Pavlova E.A., Livchitz J.L., Pels H.J., Roubtzova V.P. (1937). Researches on sandfly fever. Part VIII. Transmission of sandfly fever virus by sandflies hatched from eggs laid by infected females. Meditsinskaya Parazitologiya I Parazit. Bolezn..

[90] Petrischeva P., Alymov A. (1938). On transovarial transmission of virus of pappataci fever by sandflies. Arch. Biol. Sci..

[91] Whittingham H.E. (1924). The etiology of phlebotomus fever. Public Health.

[92] Theodor O. (1936). On the relation of *Phlebotomus papatasii* to the temperature and humidity of the environment. Bull. Entomol. Res..

[93] Tesh R.B., Chaniotis B.N. (1975). Transovarial transmission of viruses by phlebotomine sandflies. Ann. N. Y. Acad. Sci..

[94] Bartelloni P.J., Tesh R.B. (1976). Clinical and serologic responses of volunteers infected with phlebotomus fever virus (Sicilian type). Am. J. Trop. Med. Hyg..

[95] Tesh R.B., Modi G.B. (1987). Maintenance of toscana virus in *Phlebotomus perniciosus* by vertical transmission. Am. J. Trop. Med. Hyg..

[96] Tesh R.B., Chaniotis B.N., Peralta P.H., Johnson K.M. (1974). Ecology of viruses isolated from Panamanian phlebotomine sandflies. Am. J. Trop. Med. Hyg..

[97] Tesh R., Saidi S., Javadian E., Nadim A. (1977). Studies on the epidemiology of sandfly fever in Iran. I. Virus isolates obtained from Phlebotomus. Am. J. Trop. Med. Hyg..

[98] Schmidt J., Schmidt M., Said M.I. (1960). Phlebotomus fever in Egypt. Isolation of phlebotomus fever viruses from *Phlebotomus papatasi*. Am. J. Trop. Med. Hyg..

[99] Aitken T., Woodall J.P., de Andrade A., Bensabath G., Shope R.E. (1975). Pacui virus, phlebotomine flies, and small mammals in Brazil: An epidemiological study. Am. J. Trop. Med. Hyg..

[100] Tesh R.B., Modi G.B. (1984). Studies on the biology of *Phleboviruses* in sand flies (Diptera: Psychodidae) I. experimental infection of the vector. Am. J. Trop. Med. Hyg..

[101] Tesh R.B., Boshell J.S., Young D.G., Morales A.A., Corredor A.A., Modi G.B., de Carrasquilla C.F., de Rodriquez C., Gaitan M.O. (1986). Biology of arboledas virus a new phlebotomus fever serogroup virus (Bunyaviridae: *Phlebovirus*) isolated from sand flies in Colombia. Am. J. Trop. Med. Hyg..

[102] Endris R.G., Tesh R.B., Young D.G. (1983). Transovarial Transmission of Rio Grande Virus (Bunyaviridae: *Phlebovirus*) by the Sand Fly, *Lutzomyia Anthophora*. Am. J. Trop. Med. Hyg..

[103] Yu X.-J., Liang M.-F., Zhang S.-Y., Liu Y., Li J.-D., Sun Y.-L., Zhang L., Zhang Q.-F., Popov V.L., Li C. (2011). Fever with thrombocytopenia associated with a novel Bunyavirus in China. N. Engl. J. Med..

[104] Zhang Y.-Z., He Y.-W., Dai Y.-A., Xiong Y., Zheng H., Zhou D.-J., Li J., Sun Q., Luo X.-L., Cheng Y.-L. (2012). Hemorrhagic fever caused by a novel Bunyavirus in China: Pathogenesis and correlates of fatal outcome. Clin. Infect. Dis..

[105] Kim K.-H., Yi J., Kim G., Choi S.J., Jun K.I., Kim N.-H., Choe P.G., Kim N.-J., Lee J.-K., Oh M.-D. (2013). Severe fever with thrombocytopenia syndrome, South Korea, 2012. Emerg. Infect. Dis. J..

[106] Takahashi T., Maeda K., Suzuki T., Ishido A., Shigeoka T., Tominaga T., Kamei T., Honda M., Ninomiya D., Sakai T. (2014). The first identification and retrospective study of severe fever with thrombocytopenia dyndrome in Japan. J. Infect. Dis..

[107] Niu G., Li J., Liang M., Jiang X., Jiang M., Yin H., Wang Z., Li C., Zhang Q., Jin C. (2013). Severe fever with thrombocytopenia syndrome virus among domesticated animals, China. Emerg. Infect. Dis. J..

[108] Rainey T., Occi J.L., Robbins R.G., Egizi A. (2018). Discovery of *Haemaphysalis longicornis* (Ixodida: Ixodidae) parasitizing a sheep in New Jersey, United States. J. Med. Entomol..

[109] ProMED-mail (2018). Invasive tick—USA (09): (New York). http://httwww.promedmail.org/post/20180719.5915226.

[110] ProMED-mail (2018). Invasive tick—USA (11): (Pennsylvania). http://www.promedmail.org/post/20180801.5942213.

[111] Zhuang L., Sun Y., Cui X.-M., Tang F., Hu J.-G., Wang L.-Y., Cui N., Yang Z.-D., Huang D.-D., Zhang X.-A. (2018). Transmission of severe fever with thrombocytopenia syndrome virus by *Haemaphysalis longicornis* Ticks, China. Emerg. Infect. Dis..

[112] McMullan L.K., Folk S.M., Kelly A.J., MacNeil A., Goldsmith C.S., Metcalfe M.G., Batten B.C., Albariño C.G., Zaki S.R., Rollin P.E. (2012). A new phlebovirus sssociated with severe febrile illness in Missouri. N. Engl. J. Med..

[113] Pastula D.M., Turabelidze G., Yates K.F., Jones T.F., Lambert A.J., Panella A.J., Kosoy O.I., Velez J.O., Fischer M., Staples J.E. (2014). Heartland virus disease—United States, 2012–2013. Mmwr. Morb. Mortal. Wkly. Rep..

[114] Muehlenbachs A., Fata C.R., Lambert A.J., Paddock C.D., Velez J.O., Blau D.M., Staples J.E., Karlekar M.B., Bhatnagar J., Nasci R.S. (2014). Heartland virus associated death in Tennessee. Clin. Infect. Dis. Off. Publ. Infect. Dis. Soc. Am..

[115] Godsey J.M.S., Savage H.M., Burkhalter K.L., Bosco-Lauth A.M., Delorey M.J. (2016). Transmission of *Heartland Virus* (Bunyaviridae: *Phlebovirus*) by experimentally infected *Amblyomma americanum* (Acari: Ixodidae). J. Med. Entomol..

[116] Edgar R.C. (2004). MUSCLE: Multiple sequence alignment with high accuracy and high throughput. Nucleic Acids Res..

[117] Capella-Gutiérrez S., Silla-Martínez J.M., Gabaldón T. (2009). trimAl: A tool for automated alignment trimming in large-scale phylogenetic analyses. Bioinformatics.

[118] Darriba D., Taboada G.L., Doallo R., Posada D. (2011). ProtTest 3: Fast selection of best-fit models of protein evolution. Bioinformatics.

[119] Ronquist F., Teslenko M., van der Mark P., Ayres D.L., Darling A., Höhna S., Larget B., Liu L., Suchard M.A., Huelsenbeck J.P. (2012). MrBayes 3.2: Efficient Bayesian phylogenetic inference and model choice across a large model space. Syst. Boil..

[120] Raikhel A.S., Harris K.F. (1992). Vitellogenesis in Mosquitoes. Advances in Disease Vector Research.

